# Information-seeking in humans and great apes: a commentary on Kliesch (2025)

**DOI:** 10.1098/rspb.2025.1072

**Published:** 2025-07-09

**Authors:** Richard Moore

**Affiliations:** ^1^ Department of Philosophy, University of Warwick, Coventry, UK

**Keywords:** social cognition, attention, humans, great apes, secondary altriciality, information-seeking, communication

Kliesch’s postnatal dependency hypothesis argues that differences in the social attention of human and non-human great apes are a product of our social interactions in the first year of life [1]. These differences stem from humankind’s secondary altriciality, which is characterized by a unique combination of precocious cognitive development and slow-developing motor skills. Curious young chimpanzees are mobile enough to explore their environment alone. In contrast, preverbal infants undergo a period of restricted mobility that constrains their attention, leading them to focus on their caregivers. During this time, a key source of learning about the world is by attending to their caregivers’ interactions with and responses to it. These different patterns of infant attention predispose humans and great apes to different kinds of learning, which persist into later life—hypersocial in the case of humans and more individualistic in the case of great apes.

This elegant hypothesis explains key differences between our species’ communicative and socio-cognitive abilities and gestures towards an account of the origins of the unique cultural features of humankind without requiring the postulation of additional selection processes in recent hominin history. Thus, it may help us to answer a long-standing puzzle about the seemingly rapid emergence of certain distinctively human cognitive traits.

Tomasello’s classic 1999 book, *The Cultural Origins of Human Cognition* [2], opens with a puzzle. Despite sharing approximately 99% of our genetic material, the differences between humans and our closest great ape cousins seem substantial. While chimpanzees use limited toolsets, we manufacture on an industrial scale. While chimpanzees communicate using a moderate repertoire of gestures and vocalizations, we use symbolic languages, writing, mathematical notation and art. For all that some cultural behaviours are found in chimpanzee communities—patterns of tool selection or food preferences passed from one generation to the next by social learning [3]—humans inhabit institutional realities, populated by cultural traits like organized religion, hierarchical governments, a market economy, and the rule of law. Tomasello hypothesized that a fundamental cognitive change must explain these differences, and it must have emerged within a relatively compressed evolutionary timetable. While *Homo sapiens* split from our last common ancestor (LCA) with chimpanzees and bonobos approximately 7 Ma, ‘the human lineage showed no signs of anything other than typical great ape cognitive skills’ [2, pp. 2−4] until around the appearance of the Acheulean toolset approximately 2 Ma. Yet by the emergence of *H. sapiens*, within approximately 250 ka, our cognitively unique species was complete, at least in its biological foundations. Subsequently, cultural tools for cognition, like natural languages and symbolic counting systems, emerged and rapidly changed the cognition of which our ancestors were capable [4]. But, argues Tomasello, it was a fundamental biological change that enabled this new wave of cultural learning to take root.

Even granting that the numbers involved in the hominin life history vary, and that claims about how long cognitive traits take to evolve are inevitably speculative, Tomasello’s basic claim stands. Between approximately 2 Ma and approximately 250 ka, our hominin ancestors underwent a period of rapid cognitive change. On the other side of this change emerged a species far more shaped by culture than our nearest relatives. Tomasello thought the seed of this change lay in a change to our ancestors’ socio-cognitive skills: ‘a single very special form of social cognition, namely, the ability of individual organisms to understand conspecifics as beings *like themselves* who have intentional and mental lives like their own’ [2, p. 5]. This opened up new possibilities for social interaction, collaboration and faithful cultural learning—including joint attention. This is a triadic form of attention that involves coordinating attention not only to features of the world but with others’ attention to those features. Joint attention emerges in humans between 9 and 12 months and has been hypothesized to explain our language development, among other forms of collective activity. It is seemingly absent from great apes. (Chimpanzees intentionally attend to the same thing at the same time, but they seemingly do not coordinate their attention in the manner described by Tomasello [2].)

When his 1999 book was published, Tomasello still thought that great apes lack an understanding of the minds of their peers. Twenty-five years of subsequent research have shown us that chimpanzees and bonobos do understand others as intentional agents, and that their understanding of mental states is comparable to that of preverbal infants [5,6]. This does not amount to an adult-like understanding of mental states, which is probably language-dependent [4]. Still, even at the preverbal level, there are differences in the way in which humans and great apes interact with peers in their natural environments, and these have downstream effects for the development of uniquely human forms of culture.

Humans are social learners par excellence [2,7]. We copy each other’s actions at a level of detail not seen in other great ape species, and even when the reasons for doing so are difficult to fathom (‘over-imitation’). In contrast, our great ape cousins adopt more individualistic learning styles. When great apes learn to use tools by observing others, they attend primarily to mechanically effective interactions between the tools a peer is using and that peer’s environment. This allows them to grasp the functions (or ‘affordances’) of the tools and to use them in pursuit of their own goals. However, beyond basic points of contact between the observed individual and their tools, chimpanzees are less inclined to copy the tool user’s techniques [2,8]. Techniques for gripping or striking are instead learned individually, through trial and error. Humans do this too, but humans copy peers more carefully [8]. The contrast in learning styles has been described in terms of a difference between imitation and emulation [2,7]. Emulators learn about the world by watching their peers interact with it, then use what they have learned to devise their own strategies for navigating their environment. Imitators copy their peers’ actions and strategies more faithfully. Emulation is rational, but it makes chimpanzees relatively inattentive to features of interactions to which humans might orient. Consequently, emulators are likely to be poor at learning behaviours that depend on high-fidelity copying. For example, children but not chimpanzees copied the tying of a difficult knot [8]. Copying arbitrary, conventionalized sounds or movements, like the words of a language, is also difficult for chimpanzees. While an isolated individual can figure out how to use a hammer to crack a nut, the words of a language must be copied accurately from language-users to be useable [9].

These differences in social learning are mirrored in the domain of communication. Pointing comprehension has been studied using object choice tasks, in which a human experiment hides food or a prize in one of the two identical-looking boxes, before pointing to indicate the location of the hidden reward [10]. Chimpanzees raised in captive environments, but without being enculturated, are typically poor at understanding the informative points of humans—perhaps because it features only rarely in their communicative repertoire in the wild [11]. In contrast, pointing comprehension is straightforward for infants [2]. It has sometimes been argued that chimpanzees cannot understand points because they do not understand communicative intentions [10]. This explanation looks increasingly unlikely. Enculturated chimpanzees and bonobos—those raised in human environments—do understand informative pointing [12]. These findings suggest that when captive, unenculturated chimpanzees fail in standard object choice tasks it is because they are not paying attention [13].

Both chimpanzees’ pointing failures and their social learning abilities can be explained as a symptom of world-oriented, individualistic search strategies [13]. When great apes are faced with a puzzle, they first try to figure it out for themselves by studying the environment. In tool tasks, they attend to points of contact between tools and the world [2,8]. In object choice paradigms, they may look for environmental clues to the location of food—but if these are not forthcoming, chimpanzees guess (as when they fail in object choice tasks [11]). This explanation gains credibility from an eye-tracking study of great apes’ gaze-following behaviour [14]. Kano *et al.* sought to determine whether chimpanzees would follow an experimenter’s gaze to one of the two identical targets more in a communicative condition than in a non-communicative condition. Chimpanzees did not look longer at the cued target in the communicative condition. However, they spent longer examining both target objects than in the control, scanning the screen for evidence about what the experimenter might be telling them. In other words, they sought to use an individualistic search strategy to figure out the experimenter’s message rather than his gaze cues.

This finding suggests that great apes recognized the experimenter’s communicative intent but did not use his gaze cues to identify the object of his attention. Again, this strategy is rational. If one hears an indistinct cry, one can learn about its source by scanning the environment to look for the commotion. However, once one learns that peers are a rich source of information about the world, one can learn more by attending to a wider array of cues—for example, by trying to figure out whether the person shouted ‘Fire!’, ‘Thief!’ or ‘Free cake!’. Given the existence of chimpanzee cultures [3], we know that chimpanzees are not oblivious to these social cues. However, unlike humans, they seem less inclined to seek them out. More evidence for this hypothesis can be found in comparisons of the gaze patterns of human infants and chimpanzees. In observing object-involving goal-directed actions, humans, and especially infants, spend more time looking to the actor’s face than do chimpanzees [15]. We actively look to recruit social information in ways that chimpanzees do not. Again, this can be strategically valuable. Attending to an agent’s face can tell us whether their intended action was successful, how difficult or effortful it was and whether or not they are pleased with the outcome.

Kliesch’s post-natal dependency hypothesis offers a new account of why humans lean more on others to understand the world. He attributes this tendency to a key developmental difference between humans and the other great ape species—namely, our secondary altriciality. By the time great apes are cognitively developed enough to want to explore their environment, they can locomote themselves to investigate the things that intrigue them. In contrast, human minds develop faster than human bodies. It takes human infants over twice as long as chimpanzees to self-locomote effectively (8–12 months versus 3−5 months [1]). This means that for a period of human infancy, infants are curious to explore their environment but physically unable to do so. In contrast to same-aged chimpanzees, this limits infants’ capacity for individual exploration and makes their learning dependent upon the cues provided by others.

Even when young great apes are carried by their mothers, their posture sets them up to explore the world in a way different from humans. Infant great apes are typically carried either on their mother’s chest or, when slightly older, they ride on her back. This means that even when not self-locomoting, they occupy a similar perspective on the world to their mother. In contrast, human infants spend a lot of time either being carried or on their backs [1, p. 4]. Consequently, much of their early experience is spent looking at ceilings, the sky or at their caregivers’ faces, particularly in the first months of life. This early experience of faces makes the social a key point of reference for understanding the physical world. If one’s primary source of information about the world is caregivers’ responses to it, then it pays to attend to the information that caregivers’ bodies provide. Immobile infants may learn to watch others’ faces, and perhaps their hands, as a way of figuring out what is going on beyond their limited field of vision.

Kliesch’s story offers a plausible explanation of these differences between our species. The postnatal dependency hypothesis is appealing for other reasons too. Since our secondary altriciality is already established, we can use this to explain attentional differences between humans and other great ape species without postulating adaptations for social attention in the hominin lineage. So, Kliesch’s hypothesis avoids Tomasello’s puzzle about positing novel adaptations on a short evolutionary timescale [2]. Moreover, its explanatory power depends only on the retraining of a simple mechanism that is already well understood: attention. Different ontogenetic environments, driven by differences in infant mobility, push humans and great apes to adopt different learning strategies because they attend to their environment in different ways. Humans learn to depend more on others, while other great ape species learn to figure things out for themselves. This hypothesis is also consistent with an intuitive story about the role of enculturation in great apes’ development of more human-like social cognition. Through sharing an environment with us, they can learn to attend to that environment in more human-like ways and recognize the value of social cues less salient to their wild kin. This explains the human-like performance of enculturated great apes across a range of socio-cognitive tasks [13].

The postnatal dependency hypothesis suggests valuable new lines of inquiry about the role of secondary altriciality in the development of human social cognition. It can be tested empirically, too. A good place to start would be with more systematic comparisons of social attention in human and great ape infants. These studies could be enriched with the inclusion of enculturated great ape subjects.

## Data Availability

This article has no additional data
